# Adverse childhood events and self-harming behaviours among individuals in Ontario forensic system: the mediating role of psychopathy

**DOI:** 10.1186/s12888-024-05771-7

**Published:** 2024-05-01

**Authors:** Mark Mohan Kaggwa, Gary A. Chaimowitz, Bailea Erb, Heather Moulden, Sebastien Prat, Arianna Davids, Andrew T. Olagunju

**Affiliations:** 1 Department of Psychiatry and Behavioural Neurosciences, McMaster University, Hamilton, ON Canada; 2 Forensic Psychiatry Program, St. Joseph’s Healthcare, Hamilton, ON Canada; 3 Department of Psychiatry, Mbarara University of Science and Technology, Mbarara, Uganda; 4 Discipline of Psychiatry, University of Adelaide, Adelaide, SA 5000 Australia

**Keywords:** Adverse childhood events, Forensic, Mediating effect, Ontario Review Board, Psychopathy, Psychopathy checklist revised, PCL-R, And Self-harm

## Abstract

**Background:**

Adverse childhood events (ACEs), psychopathy, and self-harming behaviours are prevalent among individuals in the forensic psychiatry system. While existing literature suggests that ACEs, self-harm, and psychopathy are interrelated, little is known about the interplay of psychopathic traits in this relationship. The present study aimed to determine the mediating role of psychopathy in the relationship between ACEs and self-harming behaviours in forensic patients.

**Methods:**

This was a retrospective study of patients under the Ontario Review Board (ORB) between 2014 and 2015. In the analysis, we included patients with complete data on ACEs, self-harming behaviours, and a Psychopathy Checklist-Revised (PCL-R) score - a measure of psychopathic traits and their severity conducted during the reporting period. Mediation analysis was based on the Baron and Kenny approach, and sensitivity analysis was performed based on the types of ACEs.

**Results:**

​​​The sample population (*n* = 593) was made up of adults, with a mean age of 41.21 (± 12.35) years and were predominantly males (92.37%). While there was a partial mediating effect of psychopathy on the relationship between ACEs and incidents of self-harming behaviours in the past year, the mediation was complete in the relationship between ACEs and a lifetime history of self-harming behaviours. Following sensitivity analysis based on the types of ACE, the mediating effects were more attributed to specific ACEs, especially having experienced child abuse or having an incarcerated household member before 18 years.

**Conclusion:**

Among forensic patients in Ontario, psychopathy mediates​ ​the relationship between experiencing ACEs and engaging in self-harming behaviours. Effective intervention to mitigate self-harming behaviours in this population should consider the potential role of psychopathy, especially among individuals who have experienced ACEs involving a history of child abuse and a family who was incarcerated.

**Supplementary Information:**

The online version contains supplementary material available at 10.1186/s12888-024-05771-7.

## Introduction

The criminal justice system has consistently had a large representation of individuals with psychopathy and those who experience adverse childhood events (ACE) [1, 2]. ACEs are traumatic events (​e.g., abuse, neglect, household dysfunction, and exposure to violence) that occur before age 18 and can negatively affect physical and mental health [3]. In general, ACEs are well-known to predict a wide range of negative outcomes, such as violence, certain personality disorders, and criminogenic behaviours [4]. Previous research has reported similar prevalence rates for ACEs across correctional and forensic psychiatric populations; it has identified analogous, similar, and unique features of ACEs and their impacts on the two population groups [5].

In Canada, forensic psychiatric patients are individuals who have committed a criminal offense and are found not criminally responsible (NCR) or unfit to stand trial due to a mental disorder [6]. Compared to the general population, forensic patients have higher rates of ACEs, self-harm, as well as psychopathy​ - a condition characterized by a lack of empathy, remorse, and guilt, as well as impulsivity, antisocial behaviour, and manipulation [7]. A concise overview of relevant themes from the literature is provided below to serve as a broad background for the empirical study reported in this paper.

### ACEs and self-harming behaviours

ACEs can have a profound negative impact on an individual, particularly those involved in the criminal justice system. Evidence from the literature on forensic psychiatric patients showed that ACEs consistently predicted self-harming behaviour [5, 8]. The greater the number of ACEs, the more likely an individual would engage in self-harming conduct during adulthood [8–12]. Moreover, some studies have highlighted the importance of the various forms of ACEs (e.g., parent substance use, having a household member(s) with a mental illness, physical abuse, emotional abuse, and history of bullying) to the risk of self-harming behaviour [8, 9]. For example, emotional and sexual abuse were the most common ACEs associated with future self-harming behaviour among incarcerated females [9]. These findings highlight the variability in the detrimental effects that different types of ACEs can have on an individual’s self-harming behaviours based on the nature and severity of ACEs and the personal factors of the victims.

### ACEs and psychopathy

Research has shown that specific ACEs, such as physical abuse during childhood, are significant predictors of psychopathic traits, primarily in individuals involved in the criminal justice system [13, 14]. Closely linked is that forensic samples that present with psychopathic traits tend to have high incidences of ACEs [5], and the severity of the ACE (e.g., more severe childhood physical abuse) was positively associated with more severe psychopathic traits, specifically within the male forensic population [5].

### Psychopathy and self-harming behaviours

The relationship between psychopathy and self-harm behaviour is complex, and several studies have noted that self-harm shares a bifurcated relationship with factors 1 and 2 of the two-factor model of psychopathy [4, 15]. Factor 2 (captured by items that elicited antisocial behaviours: criminal versatility, impulsiveness, irresponsibility, poor behaviour controls, and juvenile delinquency) of the Psychopathy Checklist-Revised (PCL-R) was significantly associated with engaging in self-harming behaviours compared to Factor 1 (affective-interpersonal deficits) [16]. Similarly, self-harming behaviour was positively related to specific characteristics of psychopathy, such as high impulsivity and sensation-seeking in the forensic population [15]. Similar findings in previous reports in non-clinical samples (e.g., undergraduate students) have demonstrated an association between Factor 2 and suicidal behaviour [16]. This is most likely due to the high loading of impulsivity and antisocial tendencies in Factor 2 [15].

### Relationship between psychopathy, ACEs, and self-harming behaviours

Individuals with severe mental illness (such as those in the forensic psychiatric settings) are more prone to engage in self-harming behaviours [9, 11, 17, 18]. ACEs have been implicated as one of the plausible explanatory factors for self-harming behaviours [19]. Previous studies among forensic populations have demonstrated an increased likelihood of engaging in self-harming behaviours in individuals with a history of exposure to ACEs [5, 8] or those with psychopathic traits [15, 16]. Taking together, it is tenable to suggest that exposure to ACEs can lead to psychopathic traits, which in turn can heavily influence the prevalence of self-harming behaviour. Therefore, there is a need to explore the inter-relatedness of ACEs, psychopathy, and self-harming behaviours in the forensic population.

### Mediating effects of psychopathy on the relationship between ACEs and self-harming behaviours

While previous studies have established a link between ACEs and self-harming behaviours [5, 8], the contribution and interplay of identifiable putative factors on this relationship is yet unclear. Some theories have indicated that psychopathy (or PCL-R scores) can mediate the relationship between ACEs and self-harming behaviour [20]. One potential reason for this relationship is that when someone experiences multiple ACEs, they may develop psychopathic traits such as impulsive behaviour and a lack of emotional regulation to help cope with their situation and previous stressful circumstances or adverse experiences [13]. In turn, impulsive behaviours and antisocial tendencies are positively associated with self-harming behaviours, indicating the mediating effect of psychopathy or PCL-R scores on the risk of self-harm among individuals exposed to ACEs.

### The present study

Self-harm is a significant public health issue that can lead to severe complications, including suicide, infection, psychosocial impairment, and disability [21]. Understanding the factors associated with self-harming behaviours is a significant step toward mitigating the risks, especially among at-risk populations (e.g., individuals in the forensic system). Among forensic patients, previous studies have shown a linkage between ACEs and an increased risk of self-harming behaviours, such as cutting, burning, or hitting oneself [8–11]. Closely related is that psychopathy may influence the relationship between ACEs and self-harm by affecting emotional regulation, coping skills, and motivation for self-injury in the affected individuals [16, 22]. However, there is scant research on the mediating effects of psychopathy on the association between ACEs and self-harm among forensic patients. The present study aims to fill this gap by examining the role of psychopathy in the link between ACEs and self-harming behaviours among forensic patients. The study utilized data from individuals under the Ontario Review Board Database (ORB) in 2014 and 2015 [23]. The database was created to capture information from ORB reports for a defined period on study-specific items, including measures of ACEs, psychopathy, and self-harm [23, 24]. The study will test the hypothesis that psychopathy mediates the effect of ACEs on self-harm. Optimally, we hope that findings from the study will extend current knowledge on the etiology and prevention of self-harm among forensic patients and improve the understanding of the interplay of psychopathy on ACE and self-harm in this population. Specific hypotheses based on current literature [13, 16] are listed below.

#### Hypotheses

H1: Exposure to ACEs will be positively associated with involvement in self-harming behaviours.

H2: Exposure to ACEs will be positively correlated with psychopathy.

H3: Higher score for psychopathy will be positively associated with self-harming behaviours.

**Fig. 1:**
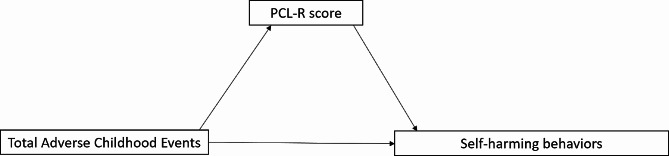
The proposed theoretical framework

H3: On the basis of the above relationships, psychopathy is likely to mediate the relationship between exposure to ACEs and involvement in self-harming behaviours (Fig. 1).

## Methods

### Study design and participants

The mediation analysis reported in this study was prepared following the Guideline for Reporting Mediation Analyses (AGReMA) [25]. We included individuals in the databases with complete data from screening with the PCL-R that resulted in scores for psychopathy for the reporting years of 2014 and 2015 (*n* = 593) [23]. Individuals in the forensic system are screened with a PCL-R based on clinical indications or the presentation of individuals, particularly those with multiple symptoms signalling psychopathy. The PCL-R is also completed as part of psycho-diagnostics and/or risk assessment for forensic patients.

### Study variables

#### Exposure (independent variable)

Adverse childhood events (ACEs) were considered as the exposure variables. Eight types of ACEs were captured (the details are provided in the study results), and each variable was dichotomized (yes/no). A yes response indicated an exposure to ACEs, and this is scored one. A response of “no’’ indicates the absence of exposure to ACEs and scored zero. The total score for all the ACEs was used to determine the severity of ACEs experienced, and the severity scores ranged between zero and eight.

#### Mediator

The Psychopathy Checklist-Revised (PCL-R) score was considered the mediator variable. PCL-R is commonly used to assess the presence of psychopathy traits in an individual [26]. The total score was captured from the ORB reports. Psychiatrists and/or psychologists trained in using the PCL-R assessed for psychopathic traits based on the tool. The total score ranges between 0 and 40, with a higher score indicating a higher risk of violence and psychopathic traits [27]. A cut-off of 30 was used to categorize individuals with psychopathy [27].

#### Outcome

The past year and lifetime history of self-harming behaviour was compiled using a variable that captured self-harm during the reporting year under the ORB system. The variable was reported as yes and no for the presence and absence of these self-harming behaviours, respectively.

#### Covariates

The covariates consist of demographic variables (age, gender, level of education, and marital status) and clinical characteristics (lifetime history of substance use, previous psychiatry hospitalization, primary psychiatric diagnosis, and presence of a comorbid psychiatric diagnosis).

### Data analysis

Data were cleaned and analysed using STATA version 16. Continuous variables were presented using means and standard deviation, while categorical variables were presented using frequencies and percentages. Inferential statistics were conducted using chi-square tests and t-tests for categorical and continuous variables, respectively. Pearson correlation coefficients were used to show the relationship between continuous variables. A *p*-value of < 0.05 was set as statistical significance with a 95% confidence interval.

Mediation analysis was based on the Baron and Kenny approach [28] with PCL-R as the mediator, self-harming behaviours as the outcomes, and the total ACEs score as the exposure. The Baron and Kenny approach is based on the following steps: Step 1 – involves regression between the exposure variable (total ACEs) with the mediating variable (PCL-R score); Step 2 – involves regression between the mediating variable and outcome variable (self-harming behaviours); step 3 – involves regression between exposure variable and outcome variable; and the Sobel’s test is conducted. Sobel’s test assesses the statistical significance of the indirect effect of the exposure and outcome through the mediator, using effect size and standard error of steps 1 and 2. If Sobel’s test is significant, then mediation is supported. However, if steps 1 or 2 are statistically significant, but Sobel’s test is not significant, the mediation is partial. Otherwise, mediation is absent. In STATA, we employed the following commands: (i) sem, (ii) estat teffects, and then (iii) medsem to test for mediation.

Sensitivity analyses for mediation effects of PCL-R on the relationship between individual types of ACEs and self-harming behaviours (both past year and lifetime) were completed. Therefore, a total of nine mediation tests were performed.

## Results

### Study sample

The data was based on individuals who had complete data on all the main variables of the study. The PCL-R score was normally distributed with a kurtosis of 2.47 and a skewness of 0.23. A total of 48 participants had no recordings of ACEs. These were denoted as missing. The remaining ACE results were normally distributed with a kurtosis of 4.01 and skewness of 1.0.

#### Clinical and sociodemographic characteristics

The mean age of the participants was 41.21 (± 12.35) years. A total of 545 (92.37%) individuals were male. Most of the participants were single (96.17%) and had an education level ranging between grades 9 and 13 (57.10%). Most included individuals were being managed for a psychotic disorder [schizophrenia and other psychotic disorders] (84.75%), used psychoactive substances (73.41%) and had a comorbid medical illness (80%). (See Table 1)

**Table 1:** Distribution of clinical and sociodemographic characteristics, and differences based on PCL-R scores, total number of ACEs, and self-harming behaviours

Variable	Overall sample *N* = 590	PCL-R score n (%)		ACEs Mean (SD)		Past year Self-harm n (%)		Lifetime self-harming behaviours	
		15.26 (7.42)	F/t-test (*p*-value)	1.22 (1.30)	t-test (*p*-value)	26 (4.43%)	χ 2 (*p*-value)	105 (17.80)	χ 2 (*p*-value)
**Sociodemographic characteristics**									
**Gender**									
Male	545 (92.37)	15.55 (7.37)	**3.24 (0.001)**	1.19 (1.31)	-1.94 (0.053)	22 (4.06)	2.43 (0.119)	91 (16.70)	**5.90 (0.015)**
Female	45 (7.63)	11.84 (7.20)		1.58 (1.08)		4 (0.09)		14 (31.11)	
**Marital status**									
Single	553 (96.17)	15.39 (7.38)	1.57 (0.117)	1.23 (1.30)	1.78 (0.075)	24 (4.36)	0.96 (0.328)	94 (17.00)	0.95 (0.329)
Married/In a common law relation	22 (3.83)	12.85 (8.28)		0.73 (0.98)		0		2 (9.09)	
**Education**									
Up to grade 8	50 (8.48)	19.78 (7.75)	**24.55 (< 0.001)**	1.5 (1.47)	**3.45 (0.032)**	4 (8.00)	5.63 (0.060)	12 (24.00)	2.91 (0.234)
Between grade 9 and 13	325 (57.10)	15.95 (7.02)		1.27 (1.35)		14 (4.35)		60 (18.46)	
Post-secondary education	200 (34.42)	12.83 (7.06)		1.04 (1.11)		5 (2.51)		29 (14.50)	
**Clinical characteristics**									
**History of substance use**									
No	155 (26.59)	11.73 (6.58)	**-7.28 (< 0.001)**	0.96 (1.11)	**-2.91 (0.004)**	8 (5.19)	0.39 (0.532)	26 (16.77)	0.08 (0.783)
Yes	428 (73.41)	16.58 (7.30)		1.31 (1.36)		17 (4.00)		76 (17.76)	
**Primary psychiatry diagnosis**									
Psychosis	500 (84.75)	14.81 (7.10)	**8.80 (< 0.001)**	1.21 (1.31)	0.45 (0.774)	19 (3.82)	**14.24 (0.007)**	85 (17.00)	**15.59 (0.004)**
Mood disorders	33 (5.59)	15.64 (7.26)		1.15 (1.23)		0		2 (6.06)	
Neurodevelopmental disorder	17 (2.88)	12.99 (8.97)		1.0 (1.17)		3 (17.65)		7 (41.18)	
Personality disorder	11 (1.89)	19.92 (7.18)		1.27 (1.10)		2 (18.18)		5 (45.45)	
Others	29 (4.92)	22.23 (8.34)		1.48 (1.35)		2 (6.90)		6 (20.69)	
**Comorbid medical illnesses**									
No	118 (20.00)	10.67 (6.56)	**-7.89 (< 0.001)**	0.89 (1.04)	**-3.08 (0.002)**	4 (3.42)	0.36 (0.550)	12 (10.17)	**5.86 (0.015)**
Yes	472 (80.00)	16.41 (7.18)		1.30 (1.35)		22 (4.69)		93 (19.70)	

#### ACEs

The average ACEs experienced were 1.22 ± 1.30. Individuals who attained lower levels of education experienced more ACEs than those with a post-secondary level of education. The use of substances of addiction was associated with experiencing significantly more ACEs than those without. Also, individuals with comorbid medical conditions experienced more ACEs than those without. (For details see, Table 1). Approximately 61.86% of the participants experienced ACEs. The most experienced ACE was child abuse (31.12%, *n* = 178), followed by a loss of a parent before 18 years (28.96%, *n* = 170), and intergenerational abuse (0.51%, *n* = 3) was the least experienced ACE (Table 2).

**Table 2:** Relationship of ACEs with PCL-R scores, psychopathy, and self-harming behaviours

Adverse childhood events	n (%)	PCL-R Mean (SD)		Psychopathy n (%)		Past year Self-harming behaviours n (%)		Lifetime Self-harming behaviours n (%)	
		15.27 (7.42)	t (*p*-value)	16 (2.71%)	t (*p*-value)	26 (4.43%)	χ2 (*p*-value)	105 (17.80)	χ2 (*p*-value)
Participants’ mothers were treated violently	70 (11.95)	17.06 (7.89)	**-2.14 (0.033)**	2 (2.86)	0.01 (0.945)	3 (4.29)	0.01 (0.937)	10 (14.29)	0.71 (0.398)
Substance abuse in the household	121 (20.75)	16.85 (7.81)	**-2.73 (0.007)**	5 (4.13)	1.48 (0.224)	9 (7.63)	3.40 (0.065)	27 (22.31)	1.91 (0.166)
Mental illness sufferers in the household	125 (21.48)	15.20 (6.88)	-0.61 (0.544)	4 (3.20)	0.25 (0.620)	9 (7.20)	2.71 (0.10)	32 (25.60)	**6.15 (0.013)**
Loss of a parent below 18 years	170 (28.96)	16.49 (0.55)	**-2.52 (0.012)**	4 (3.35)	0.12 (0.723)	7 (4.12)	0.07 (0.797)	31 (18.24)	0.02 (0.888)
Incarceration of a household member	7 (1.20)	25.49 (6.14)	**-3.70 (0.001)**	1 (14.29)	3.54 (0.060)	1 (14.29)	1.59 (0.207)	2 (28.57)	0.54 (0.463)
Intergenerational abuse	3 (0.51)	19.50 (9.85)	-0.98 (0.325)	0	0.08 (0.771)	0	0.14 (0.707)	1 (33.33)	0.49 (0.486)
Living in a foster care	44 (7.51)	19.27 (7.65)	**-4.00 (< 0.001)**	3 (6.82)	3.46 (0.063)	4 (9.09)	2.38 (0.123)	17 (38.64)	**14.22 (< 0.001)**
History of child abuse	178 (31.12)	16.66 (0.56)	**-3.35 (< 0.001)**	6 (3.37)	0.57 (0.452)	9 (5.14)	0.33 (0.565)	43 (24.16)	**7.51 (0.006)**

#### PCL-R score

The mean PCL-R score was 15.26 ± 7.42, and there were statistically significant differences in the PCL-R scores based on the study’s participants’ gender, education level, history of substance use, primary psychiatric diagnosis, and having a comorbid medical condition. That is, the score was statistically higher among males compared to females, those with lower education, who used substances, and those with comorbid medical illnesses. For details, see Table 1.

At a cut-off of 30, the prevalence of psychopathy was 7.46% (*n* = 44), and no individuals scored between 25 and 30 (a cut-off for psychopathy in some studies).

#### Self-harming behaviours

The prevalence of lifetime engagement in self-harming behaviour was 17.80% (*n* = 105). More females had proportionally engaged in self-harming behaviours in their lifetime compared to males (31.11% vs. 16.70%, χ^2^ = 5 0.90, *p*-value = 0.015). Also, individuals with comorbid medical illness had engaged more in self-harming behaviours in their lifetime (19.70% vs. 10.17%; χ^2^ = 5.86, *p*-value = 0.015). About 4.43% (*n* = 26) had self-harming behaviours over the ORB reporting years explored in this study, and among them, 19 (73.08%) had engaged in self-harming behaviours in the past year. Similar to lifetime self-harming behaviours, self-harming was significantly higher statistically among individuals with neurodevelopmental or personality disorders (Table 1).

### Relationship of ACEs with PCL-R scores, psychopathy, and self-harming behaviours

With the exception of intergeneration abuse and staying in a household with an individual having a mental illness before the age of 18, all of the other types of ACEs showed statistically significant higher mean PCL-R scores among individuals who had experienced ACEs than those who did not. There was no statistical difference between individual ACEs and psychopathy. For details, see Table 2.

Among individuals that had ever engaged in self-harming behaviour (lifetime), nine (8.57%) had psychopathy, and there were significantly more individuals with past-year self-harming behaviours without psychopathy compared to those with psychopathy (91.43% vs. 8.57%, χ2 = 7.95, *p*-value = < 0.001) statistically. Individuals who experienced the following types of ACEs, i.e., had lived in a household with an individual with mental illness below 18 years, lived in a foster home, or had experienced child abuse engaged in more lifetime self-harming behaviours on average had a higher score on PCL-R than those who did not (Table 2).

There was no statistical difference between individual types of ACEs and past year self-harming behaviours (Table 2). Among individuals with past-year self-harming behaviours, three (11.54%) had psychopathy, and there were significantly more individuals with past-year self-harming behaviours without psychopathy compared to those with psychopathy (88.46% vs. 11.54%, χ 2 = 7.95, *p*-value = 0.005) statistically.

### Correlation of PCL-R scores, total number of ACEs, and raw age

A significant positive correlation (*r* = 0.19) existed between experiencing ACEs and having a higher PCL-R score (Table 3).

**Table 3:** Correlation between PCL-R scores, total number of ACEs and age

Variables	Mean (SD)	Correlation coefficients (r)		
		PCL-R score	ACEs	Age
PCL-R score	15.27 (7.42)	1		
ACEs	1.22 (1.30)	0.19 **	1	
Age	41.21 (12.35)	-0.05	-0.06	1

### Testing the mediating effect of PCL-R on the relationship between ACEs and self-harming behaviours

#### Past year self-harming behaviours

In step 1, ACEs were significantly associated with PCL-R scores (*β* = 1.085, *p*-value = < 0.001). In step 2, the PCL-R score was significantly associated with past year self-harming behaviours (*β* = -0.003, *p*-value = 0.005). However, in step 3, ACEs were not significantly associated with past year self-harming behaviours (*β* = 0.007, *p*-value = 0.294). As steps 1, 2, and Sobel’s test are significant, but step 3 is not significant, the mediation is complete. (Supplementary Table 1).

**Fig. 2:**
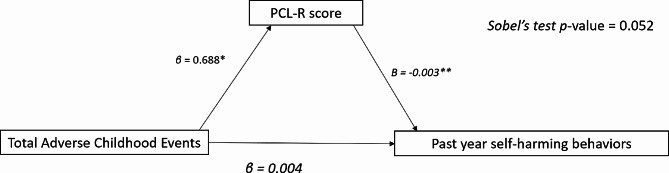
Mediating role of PCL-R between ACEs and past year self-harming behaviours

After controlling for clinical and sociodemographic factors, Step 1 showed that ACEs were significantly associated with the PCL-R scores (*β* = 0.680, *p*-value = 0.002). In step 2, the PCL-R score was significantly associated with past year self-harming behaviours (*β* = 0.003, *p*-value = 0.012). However, in step 3, ACEs were not significantly associated with past year self-harming behaviours (*β* = 0.004, *p*-value = 0.557). As steps 1 and 2 are significant, and neither step 3 nor Sobel’s test of the indirect effect was significant (0.002, *p*-value = 0.052), the mediation of PCL-R between ACEs and past year self-harming behaviour is partial. (Fig. 2 and Supplementary Table 1.

#### Lifetime self-harming behaviours

In step 1, ACEs were significantly associated with the PCL-R scores (*β* = 1.091, *p*-value < 0.001). In step 2, the PCL-R score was significantly associated with lifetime self-harming behaviours (*β* = 0.003, *p*-value < 0.001). In step 3, ACEs were also significantly associated with lifetime self-harming behaviours (*β* = 0.026, *p*-value = 0.033). The mediation is partial as steps 1, 2, 3, and Sobel’s test are significant. (Supplementary Table 2).

**Fig. 3:**
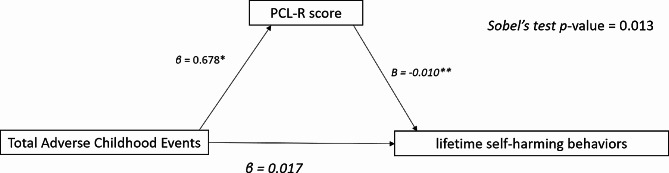
Mediating role of PCL-R between ACEs and lifetime self-harming behaviours

After controlling for clinical and sociodemographic factors, in Step 1, ACEs were significantly associated with the PCL-R scores (*β* = 0.678, *p*-value = 0.002). In step 2, the PCL-R score was significantly associated with lifetime self-harming behaviours (*β* = 0.010, *p*-value < 0.001). However, in step 3, ACEs were not significantly associated with lifetime self-harming behaviours (*β* = 0.017, *p*-value = 0.167). As steps 1, 2, and Sobel’s test (0.01, *p*-value = 0.013) are significant, but step 3 was not significant, the mediation of PCL-R between ACEs and lifetime self-harming behaviour is complete. (Fig. 3 and Supplementary Table 2).

### Sensitivity analysis for mediation effect of PCL-R score on the relationships of total and types of ACEs with self-harm behaviours

The mediating effect of the PCL-R score for the total ACEs almost mirrored that of individuals who had experienced child abuse and incarceration of a household member. (See Table 4). Complete mediation was observed among those with lifetime self-harm and having a history of child abuse or a household member incarcerated. The details of the sensitivity analysis are presented in Supplementary Tables 1 and 2.

**Table 4:** Summary of mediation effects of PCL-R on the relationships of total and types of ACEs with self-harming behaviours

ACEs	Self-harming behaviours	Mediation	
		unadjusted	Adjusted
Total ACEs	Past year	Complete	Partial
	Lifetime	Partial	Complete
Mother treated violently	Past year	Partial	No
	Lifetime	Partial	No
Substance use in household	Past year	Complete	No
	Lifetime	Complete	No
Mental illness sufferer in household	Past year	No	No
	Lifetime	No	No
Loss of a parent below 18 years	Past year	Complete	No
	Lifetime	Complete	No
Incarceration of a household member	Past year	Complete	Partial
	Lifetime	Complete	Complete
Intergeneration abuse	Past year	No	No
	Lifetime	No	No
Living in foster care	Past year	Complete	Partial
	Lifetime	Partial	Partial
History of child abuse	Past year	Complete	Partial
	Lifetime	Partial	Complete

## Discussion

### Overview of the study findings

The present study found a partial mediating effect of psychopathy on the relationship between ACEs and past-year self-harming behaviours. However, the mediation effect was complete in relation to lifetime self-harming behaviours. Overall, the mediating effect of psychopathy on the relationship between total ACEs and self-harming behaviours almost mirrored that of individuals who had experienced child abuse and incarceration of a household member. Other interesting findings from the study and the implications are discussed below.

### Prevalence of psychopathy, distribution of PCL-R score, and the associated factors

Out of the 590 eligible individuals who were included, approximately 7.49% had psychopathy based on a cut-off score of 30. The prevalence rate in the present study is higher than the pooled prevalence rate of 1.2% reported in a meta-analytic review of studies conducted among the general population using the same tool and cut-off score [29]. However, the prevalence reported in the current study is lower than the pooled prevalence of 27.8% for psychopathy from studies conducted among individuals in the correctional system charged with homicide [25]. The differences in the rates of psychopathy between our study and the cited studies may be attributed to the differences in the characteristics of the study populations. For example, it is possible that forensic patients (included in our study) are individuals most likely to be diagnosed primarily with severe mental illness [19], and fewer of them may have psychopathy compared to offenders involved in homicide. Similarly, a lower PCL-R mean score was observed in our study participants compared to individuals convicted of homicide (15.26 ± 7.42 vs. 21.2 ± 5.3) [25]. While the prevalence of psychopathy in our study is lower compared to the correctional population with homicide, the results were close to those of the general population [7, 29].

In keeping with the findings documented in previous meta-analytic studies, the mean score of the measure (PCL-R) for psychopathy in the present study was higher among males than females [29, 30]. A detailed explanation for this difference has been described by Beryl et al. [30]. The present study also found that the average PCL-R scores decreased with an increase in the level of education. This may be attributed to the idea that antisocial behaviours, disregard for social norms, and impulsive behaviours that are associated with psychopathy may lead to poor academic performance and, in turn, lower academic achievements [31, 32]. In addition, psychopathic characteristics may lead to higher chances of involvement with the criminal justice system, which may negatively affect an individual’s progress in school. Contrary findings have been recorded for certain professions, especially in business, where individuals with higher mean scores on the PCL-R were high academic achievers [31, 33]. The mean score for psychopathy was also higher among individuals with two interlinked conditions, i.e., substance use history and comorbid medical conditions [34], a relationship that may be attributed to the complicated lifestyle (e.g., not adhering to rules and instruction, such as failure to stay away from dangerous substances or follow medication adherence often adopted by individuals with higher psychopathic traits.)

### Prevalence of ACEs and the associated factors

Over 60% of the study participants experienced ACEs, with most experiencing child abuse. The high prevalence rate of ACEs in the present study is similar to the findings among forensic populations in other parts of the globe, such as Sweden (57.2%) [8], USA (79.4%) [35], and UK (82.8%) [9]. It is important to note that the average number of reported ACEs events (1.22±1.30) was lower in this sample than in previous studies that employed the same method of identifying ACEs, such as 2.63±2.3 among a sample of 157 forensic psychiatric patients from the USA [36]. The difference may be attributed to the smaller number of ACEs identified in the current study (8), while many studies identify more.

The mean for the total number of ACEs experienced decreased with an increase in the education level, a finding consistent with other previous studies [37]. A plausible explanation may be that ACEs have been linked with impairment of cognitive function, working memory, attention, and language acquisition, which can lead to poorer academic performance [38]. However, it is important to note that some studies have reported no significant impact of ACEs on academic performance, which are findings attributed to individuals’ resilience and protective factors [39]. Similar to individuals who scored high on PCL-R, those with a higher mean number for ACEs had a history of substance use and suffered from a comorbid medical condition.

In the present study, an increase in ACEs correlated positively with PCL-R score. Existing literature consistently reported a link between ACEs and psychopathy [13]. These findings further support the notion that a high number of individuals with ACEs are more likely to have a significantly higher PCL-R score, except for individuals with ACE resulting specifically from intergeneration abuse and staying in a household with an individual diagnosed with mental illness before the age of 18 in this study.

### Prevalence of self-harming behaviours and the associated factors

Among the study participants, approximately 5% had self-harming behaviours during the reporting years under study. This prevalence is several folds lower than reported in other forensic settings, including Sweden, the USA, and the UK, with prevalence ranging between 36.0% and 68.4% [9, 11, 17, 18, 40]. The low prevalence in the present study may be attributed to the nature of the sample population, made up mainly of individuals with psychopathy based on PCL-R evaluation. By practice, not every forensic psychiatric patient in Ontario is assessed using a PCL-R. Those deemed with high suspicion of having psychopathy get assessed, thus skewing the number that are more likely to screen positive for psychopathy or score highly on the PCL-R. These individuals with higher scores may score highly on both Factor 1 and 2 of the PCL-R. With individuals who met the criteria for psychopathy in the present study having experienced fewer incidences of self-harming behaviours than those who didn’t. We speculate that the influence of scoring highly on the specific PCL-R items that load on factor 1 (i.e., involving items related to interpersonal and affective deficits of psychopathy, including shallow affect, superficial charm, manipulativeness, lack of empathy), which are associated with less self-harming behaviours [16] led to the lower prevalence observed.

### Mediating role of PCL-R score on the effect of ACEs on self-harming behaviours

The present study found a partial mediation role of PCL-R score on the effects of total ACEs on past year self-harming behaviours after controlling for other covariates. This indicates that other variables may be explanatory of the effects of ACEs on self-harming behaviour in addition to PCL-R score, such as biological factors like inflammation [41], an aspect that is outside the scope of the present study. Consequently, further research is warranted to fully understand the interplay of psychopathic traits and other putative factors on the relationship of ACEs with self-harming behaviours among forensic patients. Again, the partial mediation may be due to the tool used (i.e., PCL-R), which may not capture all aspects of psychopathy or personality that are relevant to self-harm. For example, some researchers have argued that the PCL-R may not be adequate to measure affective and interpersonal dimensions of psychopathy, such as callousness, narcissism, or Machiavellianism, that may relate to self-harm [42]. On the other hand, the mediating relationship of PCL-R on the effects of ACEs on self-harming may potentially be since individuals who have experienced ACEs may develop psychopathic traits as a maladaptive coping mechanism [13]. The psychopathic traits (captured by the PCL-R) may, in turn, increase the likelihood of engaging in self-harming behaviours as a form of emotional regulatory mechanism or to exert control [16, 22].

Based on sensitivity analysis, psychopathy loaded higher as a mediator for self-harming behaviours for individuals with ACEs from living in a foster house, having a family member previously incarcerated, and having a history of child abuse. These findings may be explained by several factors, including inherited gene influence (genes that influence psychopathy and or involvement in self-harming behaviours), adopting of maladaptive coping style, and vulnerability index.

Our study findings among individuals with a family member incarcerated before 18 years may be related to the interplay of genetics (inheritance) and learning of maladaptive coping strategies the family member who ended up incarcerated used. This nature and nurture effect may lead to using self-harming behaviours as a coping skill, developing psychopathic traits, and ending up within the correctional justice system. Research has implicated genetic links for psychopathy among multiple family members [43].

Individuals who stay in a foster home may be exposed to various forms of childhood trauma (e.g., child abuse, neglect, instabilities, etc.) that may impact their emotional development and attachment security [44]. Consequently, they are vulnerable to developing emotional dysregulation and psychopathic traits (such as lack of empathy, remorse, or guilt) that are precursors for risky behaviours [20]. Due to the emotional dysregulation and inadequate development of coping skills among these children, some may use self-harming behaviours to cope with negative emotions, express anger or frustration, seek attention or validation, or manipulate others [20]. In addition, individuals who go through the foster care system may have poor social support and limited access to quality mental health services for children. Implicitly, they are isolated, helpless, and hopeless, and engaging in self-harming behaviours becomes more likely as a coping mechanism. There are several potential explanations for the complete mediating effect of psychopathy on the linkage between being in foster care and self-harming behaviours. For example, some individuals in foster care may have brain damage from encountering severe life experiences while in the system [44] and develop psychopathic traits [45] that increase their vulnerability to engage in self-harming behaviours [46].

### Limitations

The following limitations should be considered in interpreting these study findings: (1) the individual facets of the PCL-R were not captured and used in the current analysis despite their strong and unique relationship with the variables assessed. Future studies should explore the interplay of the PCL-R facets on the relationship of ACEs with self-harming behaviours so that a targeted approach can be designed to mitigate the effects of such specific items as part of the interventions to reduce self-harming behaviours; (2) Self-harm was based on witnessed and reported incidents. This may be affected by the quality of information captured in the ORB report, and under-reporting of the incidents is possible; (3) The cross-sectional study design also limits inferences on causality, and a more robust prospective design should be employed in future studies, and (4) There is the likelihood of the introduction of systematic bias in the study since the individuals who are selected to have a PCL-R are dependent on clinician judgment, institutional policy, or requirement for ORB annual hearing. These may leave out some individuals who may score differently on the PCL-R, potentially leading to an altered picture of the mediating relationship captured. Lastly, despite the popularity of the use of the PCL-R tool among forensic psychiatry patients in Ontario, no available data has validated its use among patients with antisocial personality disorder, whose presentation and etiology may be similar to psychopathy [47]. Yet, they may pose varying risks of self-harming or a history of having been exposed to ACEs.

### Conclusions

Among forensic patients in Ontario, psychopathy plays a mediating role in the effects of ACEs on engaging in self-harming behaviours. This role is experienced mainly by individuals who had ACEs involving child abuse, incarceration of a household member, and having lived in a foster home. For effective intervention to reduce self-harming behaviours, adequate attention should be given to the effects of identifiable mediators. Further studies are recommended to explore the interplay of specific factors or items of PCL-R on the risk attributable to ACEs for incidents of self-harming behaviours in the forensic population.

### Electronic supplementary material

Below is the link to the electronic supplementary material.

Supplementary Material 1

Supplementary Material 2

## Data Availability

Due to the sensitivity of the population involved, the datasets will be made available to appropriate academic parties on request from the corresponding author after approval by Dr. Gary Andrew Chaimowitz.
