# Supplementary material for: Adverse childhood events and self-harming behaviours among individuals in Ontario forensic system: the mediating role of psychopathy
Source: BMC Psychiatry. 2024 May 1;24:332. doi: 10.1186/s12888-024-05771-7 (PMC11064378; doi:10.1186/s12888-024-05771-7)
Supplement: Supplementary file 1 — Supplementary Material 1 [file 12888_2024_5771_MOESM1_ESM.docx]

**Supplementary file 1: Mediating effects of PCL-R between ACEs and past year self-harming behaviors based on the Baron and Kenny approach**

| Variable | | Direct | | Indirect effect | | Total effect | | Sobel’s test |
| --- | --- | --- | --- | --- | --- | --- | --- | --- |
|  |  | Coef (*S.E*) | *p*-value | Coef (*S.E*) | *p*-value | Coef (*S.E*) | *p*-value |  |
| Unadjusted relationship (total number of ACEs) | | | | | | | | |
| PCL-R | ACEs | 1.08 (0.23) | <0.001 | No path |  | 1.08 (0.23) | <0.001 | 0.004 (0.001)  P-value =0.015 |
| Past year self-harming behavior | PCL-R | 0.003 (0.001) | 0.005 | No path |  | 0.003 (0.001) | 0.005 |  |
|  | ACEs | 0.007 (0.007) | 0.294 | 0.004 (0.001) | 0.015 | 0.010 (0.006) | 0.108 |  |
| Adjusted relationship ((total number of ACEs)) | | | | | | | | |
| PCL-R | ACEs | 0.68 (0.22) | 0.002 | No path |  | 0.68 (0.22) | 0.012 | 0.002 (0.001)  p-value = 0.052 |
| Past year self-harming behavior | PCL-R | 0.003 (0.001) | 0.012 | No path |  | 0.03 (0.001) | 0.012 |  |
|  | ACEs | -0.004 (0.006) | 0.557 | 0.002 (0.001) | 0.052 | -0.002 (0.006) | 0.794 |  |
| Unadjusted (Participants’ mothers were treated violently) | | | | | | | | |
| PCL-R | Participants’ mothers were treated violently | 2.002 (0.945) | 0.034 | No path |  | 2.002 (0.946) | 0.034 | 0.007 (0.004)  p-value = 0.081 |
| Past year self-harming behavior | PCL-R | 0.003 (0.001) | 0.002 | No path |  | 0.003 (0.001) | 0.002 |  |
|  | Participants’ mothers were treated violently | 0.009 (0.026) | 0.727 | -0.002 (0.026) | 0.937 | -0.002 (0.026) | 0.937 |  |
| Adjusted (Participants’ mothers were treated violently) | | | | | | | | |
| PCL-R | Participants’ mothers were treated violently | 1.195 (0.861) | 0.165 | No path |  | 1.195 (0.861) | 0.165 | 0.003 (0.003)  p-value = 0.229 |
| Past year self-harming behavior | PCL-R | 0.003 (0.002) | 0.016 | Mo path |  | 0.003 (0.002) | 0.016 |  |
|  | Participants’ mothers were treated violently | 0.001 (0.024) | 0.968 | 0.003 (0.003) | 0.229 | 0.004 (0.024) | 0.855 |  |
| Unadjusted (Substance abuse in the household) | | | | | | | | |
| PCL-R | Substance abuse in the household | 2.093 (0.759) | 0.006 | No path |  | 2.093 (0.759) | 0.006 | 0.007 (0.004)  P value = 0.044 |
| Past year self-harming behavior | PCL-R | 0.003 (0.001) | 0.003 | No path |  | 0.003 (0.001) | 0.003 |  |
|  | Substance abuse in the household | 0.032 (0.021) | 0.130 | 0.007 (0.003) | 0.044 | 0.039 (0.021) | 0.064 |  |
| Adjusted (Substance abuse in the household) | | | | | | | | |
| PCL-R | Substance abuse in the household | 0.599 (0.721) | 0.407 | No path |  | 0.599 (0.721) | 0.407 | 0.002 (0.002)  p-value = 0.432 |
| Past year self-harming behavior | PCL-R | 0.003 (0.001) | 0.016 | No path |  | 0.003 (0.001) | 0.016 |  |
|  | Substance abuse in the household | 0.005 (0.201) | 0.785 | 0.002 (0.002) | 0.432 | 0.007 (0.202) | 0.721 |  |
| Unadjusted (Mental illness sufferers in the household) | | | | | | | | |
| PCL-R | Mental illness sufferers in the household | 0.428 (0.747) | 0.567 | No path |  | 0.428 (0.747) | 0.567 | 0.002 (0.003)  p-value = 0.573 |
| Past year self-harming behavior | PCL-R | 0.004 (0.001) | 0.002 | No path |  | 0.004 (0.001) | 0.002 |  |
|  | Mental illness sufferers in the household | 0.033 (0.021) | 0.112 | 0.001 (0.002) | 0.573 | 0.034 (0.021) | 0.099 |  |
| Adjusted (Mental illness sufferers in the household) | | | | | | | | |
| PCL-R | Mental illness sufferers in the household | 0.470 (0.689) | 0.495 | No path |  | 0.470 (0.689) | 0.495 | 0.001 (0.002)  p-value = 0.512 |
| Past year self-harming behavior | PCL-R | 0.003 (0.001) | 0.017 | No path |  | 0.003 (0.001) | 0.017 |  |
|  | Mental illness sufferers in the household | 0.030 (0.019) | 0.115 | 0.001 (0.002) | 0.512 | 0.031 (0.019) | 0.017 |  |
| Unadjusted (Loss of a parent below 18 years) | | | | | | | | |
| PCL-R | Loss of a parent below 18 years | 1.674 (0.674) | 0.013 | No path |  | 1.674 (0.675) | 0.013 | 0.006 (0.003)  p-value = 0.052 |
| Past year self-harming behavior | PCL-R | 0.004 (0.001) | 0.002 | No path |  | 0.004 (0.001) | 0.002 |  |
|  | Loss of a parent below 18 years | -0.011 (0.019) | 0.565 | 0.006 (0.003) | 0.052 | -0.005 (0.019) | 0.797 |  |
| Adjusted (Loss of a parent below 18 years) | | | | | | | | |
| PCL-R | Loss of a parent below 18 years | 1.088 (0.629) | 0.084 | No path |  | 1.088 (0.629) | 0.084 | 0.003 (0.002)  p-value = 0.155 |
| Past year self-harming behavior | PCL-R | 0.003 (0.001) | 0.012 | No path |  | 0.003 (0.001) | 0.012 |  |
|  | Loss of a parent below 18 years | -0.022 (0.018) | 0.212 | 0.003 (0.002) | 0.155 | -0.019 (0.018) | 0.289 |  |
| Unadjusted (Incarceration of a household member) | | | | | | | | |
| PCL-R | Incarceration of a household member | 10.33 (2.80) | <0.001 | No path |  | 10.33 (2.799) | <0.001 | 0.035 (0.015)  p-value = 0.022 |
| Past year self-harming behavior | PCL-R | 0.003 (0.001) | 0.003 | No path |  | 0.003 (0.001) | 0.003 |  |
|  | Incarceration of a household member | 0.064 (0.079) | 0.416 | 0.035 (0.015) | 0.022 | 0.099 (0.079) | 0.207 |  |
| Adjusted (Incarceration of a household member) | | | | | | | | |
| PCL-R | Incarceration of a household member | 6.779 (2.546) | 0.008 | No path |  | 6.779 (2.546) | 0.008 | 0.019 (0.011)  p-value = 0.082 |
| Past year self-harming behavior | PCL-R | 0.003 (0.001) | 0.022 | No path |  | 0.003 (0.001) | 0.022 |  |
|  | Incarceration of a household member | 0.077 (0.071) | 0.284 | 0.019 (0.011) | 0.082 | 0.095 (0.072) | 0.182 |  |
| Unadjusted (Intergenerational abuse) | | | | | | | | |
| PCL-R | Intergenerational abuse | 4.223 (4.312) | 0.327 | No path |  | 4.223 (4.312) | 0.327 | 0.015 (0.016)  p-value = 0.350 |
| Past year self-harming behavior | PCL-R | 0.003 (0.001) | 0.002 | No path |  | 0.003 (0.001) | 0.002 |  |
|  | Intergenerational abuse | -0.060 (0.118) | 0.614 | 0.015 (0.016) | 0.350 | -0.045 (0.120) | 0.707 |  |
| Adjusted (Intergenerational abuse) | | | | | | | | |
| PCL-R | Intergenerational abuse | 1.608 (3.849) | 0.676 | No path |  | 1.608 (3.849) | 0.676 | 0.005 (0.011)  p-value = 0.681 |
| Past year self-harming behavior | PCL-R | 0.003 (0.001) | 0.015 | No path |  | 0.003 (0.001) | 0.015 |  |
|  | Intergenerational abuse | -0.043 (0.107) | 0.687 | 0.005 (0.113) | 0.681 | -0.385 (0.108) | 0.721 |  |
| Unadjusted (Living in a foster care) | | | | | | | | |
| PCL-R | Living in a foster care | 4.559 (1.147) | <0.001 | No path |  | 4.559 (1.147) | <0.001 | 0.015 (0.007)  p-value = 0.020 |
| Past year self-harming behavior | PCL-R | 0.003 (0.001) | 0.004 | No path |  | 0.003 (0.001) | 0.004 |  |
|  | Living in a foster care | 0.035 (0.032) | 0.285 | 0,015 (0.006) | 0.020 | 0.050 (0.032) | 0.122 |  |
| Adjusted (Living in a foster care) | | | | | | | | |
| PCL-R | Living in a foster care | 2.440 (1.159) | 0.035 | No path |  | 2.440 (1.159) | 0.035 | 0.008 (0.005)  p-value = 0.099 |
| Past year self-harming behavior | PCL-R | 0.003 (0.001) | 0.008 | No path |  | 0.003 (0.001) | 0.008 |  |
|  | Living in a foster care | -0.068 (0.032) | 0.037 | 0.008 (0.005) | 0.099 | 0.060 (0.033) | 0.065 |  |
| Unadjusted (History of child abuse) | | | | | | | | |
| PCL-R | History of child abuse | 2.231 (0.665) | 0.001 | No path |  | 2.231 (0.665) | 0.001 | 0.008 (0.004)  p-value = 0.023 |
| Past year self-harming behavior | PCL | 0.004 (0.001) | 0.002 | No path |  | 0.004 (0.001) | 0.002 |  |
|  | History of child abuse | 0.003 (0.019) | 0.887 | 0.008 (0.003) | 0.023 | 0.011 (0.019) | 0.565 |  |
| Adjusted (History of child abuse) | | | | | | | | |
| PCL-R | History of child abuse | 1.747 (0.636) | 0.006 | No path |  | 1.747 (0.636) | 0.006 | 0.006 (0.003)  p-value = 0.058 |
| Past year self-harming behavior | PCL | 0.003 (0.001) | 0.009 | No path |  | 0.003 (0.001) | 0.009 |  |
|  | History of child abuse | -0.026 (0.018) | 0.151 | 0.006 (0.003) | 0.058 | -0.020 (0.018) | 0.260 |  |
