# Supplementary material for: Adverse childhood events and self-harming behaviours among individuals in Ontario forensic system: the mediating role of psychopathy
Source: BMC Psychiatry. 2024 May 1;24:332. doi: 10.1186/s12888-024-05771-7 (PMC11064378; doi:10.1186/s12888-024-05771-7)
Supplement: Supplementary file 2 — Supplementary Material 2 [file 12888_2024_5771_MOESM2_ESM.docx]

**Supplementary file 2: Mediating effects of PCL-R between ACEs and lifetime self-harming behaviors based on the Baron and Kenny approach**

| Variable | | Direct | | Indirect effect | | Total effect | | Sobel’s test p-value |
| --- | --- | --- | --- | --- | --- | --- | --- | --- |
|  |  | Coef (*S.E*) | *p*-value | Coef (*S.E*) | *p*-value | Coef (*S.E*) | *p*-value |  |
| Unadjusted relationship (total number of ACEs) | | | | | | | | |
| PCL-R | ACEs | 1.091 (0.231) | <0.001 | No path |  | 1.091 (0.231) | <0.001 | 0.002 |
| Lifetime self-harming behavior | PCL-R | 0.009 (0.002) | <0.001 | No path |  | 0.009 (0.002) | <0.001 |  |
|  | ACEs | 0.026 (0.012) | 0.033 | 0.010 (0.003) | 0.002 | 0.035 (0.012) | 0.003 |  |
| Unadjusted (Participants’ mothers were treated violently) | | | | | | | | |
| PCL-R | Participants’ mothers were treated violently | 2.024 (0.943) | 0.032 | No path |  | 2.024 (0.943) | 0.032 | 0.051 |
| Lifetime self-harming behavior | PCL-R | 0.010 (0.002) | <0.001 | No path |  | 0.010 (0.002) | <0.001 |  |
|  | Participants’ mothers were treated violently | -0.061 (0.048) | 0.203 | 0.020 (0.010) | 0.051 | -0.041 (0.049) | 0.398 |  |
| Adjusted (Participants’ mothers were treated violently) | | | | | | | | |
| PCL-R | Participants’ mothers were treated violently | 1.201 (0.859) | 0.162 | No path |  | 1.201 (0.859) | 0.162 | 0.182 |
| Lifetime self-harming behavior | PCL-R | 0.010 (0.002) | <0.001 | No path |  | 0.010 (0.002) | <0.001 |  |
|  | Participants’ mothers were treated violently | -0.058 (0.046) | 0.212 | 0.012 (0.009) | 0.182 | -0.046 (0.047) | 0.334 |  |
| Unadjusted (Substance abuse in the household) | | | | | | | | |
| PCL-R | Substance abuse in the household | 2.048 (0.750) | 0.006 | No path |  | 2.048 (0.750) | 0.006 | 0.019 |
| Lifetime self-harming behavior | PCL-R | 0.01 (0.002) | <0.001 | No path |  | 0.01 (0.002) | <0.001 |  |
|  | Substance abuse in the household | 0.034 (0.039) | 0.377 | 0.02 (0.01) | 0.019 | 0.054 (0.039) | 0.166 |  |
| Adjusted (Substance abuse in the household) | | | | | | | | |
| PCL-R | Substance abuse in the household | 0.563 (0.713) | 0.429 | No path |  | 0.563 (0.713) | 0.429 | 0.437 |
| Lifetime self-harming behavior | PCL-R | 0.01 (0.002) | <0.001 | No path |  | 0.01 (0.002) | <0.001 |  |
|  | Substance abuse in the household | 0.011 (0.038) | 0.779 | 0.006 (0.007) | 0.437 | 0.017 (0.039) | 0.673 |  |
| Unadjusted (Mental illness sufferers in the household) | | | | | | | | |
| PCL-R | Mental illness sufferers in the household | 0.453 (0.745) | 0.543 | No path |  | 0.453 (0.745) | 0.543 | 0.546 |
| Lifetime self-harming behavior | PCL-R | 0.010 (0.002) | <0.001 | No path |  | 0.010 (0.002) | <0.001 |  |
|  | Mental illness sufferers in the household | 0.092 (0.038) | 0.015 | 0.005 (0.008) | 0.546 | 0.096 (0.039) | 0.013 |  |
| Adjusted (Mental illness sufferers in the household) | | | | | | | | |
| PCL-R | Mental illness sufferers in the household | 0.480 (0.686) | 0.484 | No path |  | 0.480 (0.686) | 0.484 | 0.490 |
| Lifetime self-harming behavior | PCL-R | 0.010 (0.002) | <0.001 | No path |  | 0.010 (0.002) | <0.001 |  |
|  | Mental illness sufferers in the household | 0.109 (0.037) | 0.003 | 0.005 (0.007) | 0.490 | 0.113 (0.037) | 0.003 |  |
| Unadjusted (Loss of a parent below 18 years) | | | | | | | | |
| PCL-R | Loss of a parent below 18 years | 1.698 (0.672) | 0.012 | No path |  | 1.698 (0.672) | 0.012 | 0.027 |
| Lifetime self-harming behavior | PCL-R | 0.010 (0.002) | <0.001 | No path |  | 0.010 (0.002) | <0.001 |  |
|  | Loss of a parent below 18 years | -0.012 (0.034) | 0.736 | 0.016 (0.007) | 0.027 | 0.005 (0.035) | 0.888 |  |
| Adjusted (Loss of a parent below 18 years) | | | | | | | | |
| PCL-R | Loss of a parent below 18 years | 1.096 (0.627) | 0.080 | No path |  | 1.096 (0.627) | 0.080 | 0.104 |
| Lifetime self-harming behavior | PCL-R | 0.010 (0.002) | <0.001 | No path |  | 0.010 (0.002) | <0.001 |  |
|  | Loss of a parent below 18 years | -0.016 (0.034) | 0.643 | 0.011 (0.007) | 0.104 | -0.005 (0.035) | 0.894 |  |
| Unadjusted (Incarceration of a household member) | | | | | | | | |
| PCL-R | Incarceration of a household member | 10.352 (2.793) | <0.001 | No path |  | 10.352 (2.793) | <0.001 | 0.004 |
| Lifetime self-harming behavior | PCL-R | 0.010 (0.002) | <0.001 | No path |  | 0.010 (0.002) | <0.001 |  |
|  | Incarceration of a household member | 0.006 (0.145) | 0.966 | 0.101 (0.035) | 0.004 | 0.107 (0.146) | 0.463 |  |
| Adjusted (Incarceration of a household member) | | | | | | | | |
| PCL-R | Incarceration of a household member | 6.791 (2.540) | 0.007 | No path |  | 6.791 (2.540) | 0.007 | 0.023 |
| Lifetime self-harming behavior | PCL-R | 0.010 (0.002) | <0.001 | No path |  | 0.010 (0.002) | <0.001 |  |
|  | Incarceration of a household member | 0.026 (0.139) | 0.854 | 0.069 (0.030) | 0.023 | 0.094 (0.140) | 0.502 |  |
| Unadjusted (Intergenerational abuse) | | | | | | | | |
| PCL-R | Intergenerational abuse | 4.244 (4.304) | 0.324 | No path |  | 4.244 (4.304) | 0.324 | 0.335 |
| Lifetime self-harming behavior | PCL-R | 0.010 (0.002) | <0.001 | No path |  | 0.010 (0.002) | <0.001 |  |
|  | Intergenerational abuse | 0.114 (0.218) | 0.602 | 0.041 (0.042) | 0.335 | 0.155 (0.222) | 0.486 |  |
| Adjusted (Intergenerational abuse) | | | | | | | | |
| PCL-R | Intergenerational abuse | 1.620 (3.839) | 0.673 | No path |  | 1.620 (3.839) | 0.673 | 0.674 |
| Lifetime self-harming behavior | PCL-R | 0.010 (0.002) | <0.001 | No path |  | 0.010 (0.002) | <0.001 |  |
|  | Intergenerational abuse | 0.139 (0.207) | 0.503 | 0.016 (0.039) | 0.674 | 0.155 (0.211) | 0.462 |  |
| Unadjusted (Living in a foster care) | | | | | | | | |
| PCL-R | Living in a foster care | 4.579 (1.144) | <0.001 | No path |  | 4.579 (1.144) | <0.001 | 0.005 |
| Lifetime self-harming behavior | PCL-R | 0.010 (0.002) | <0.001 | No path |  | 0.010 (0.002) | <0.001 |  |
|  | Living in a foster care | 0.188 (0.059) | 0.001 | 0.038 (0.013) | 0.005 | 0.008 (0.002) | <0.001 |  |
| Adjusted (Living in a foster care) | | | | | | | | |
| PCL-R | Living in a foster care | 2.451 (1.156) | 0.034 | No path |  | 2.451 (1.156) | 0.034 | 0.061 |
| Lifetime self-harming behavior | PCL-R | 0.010 (0.002) | <0.001 | No path |  | 0.010 (0.002) | <0.001 |  |
|  | Living in a foster care | 0.172 (0.053) | 0.006 | 0.022 (0.012) | 0.022 | 0.194 (0.063) | 0.002 |  |
| Unadjusted (History of child abuse) | | | | | | | | |
| PCL-R | History of child abuse | 2.213 (0.660) | 0.001 | No path |  | 2.213 (0.660) | 0.001 | 0.007 |
| Lifetime self-harming behavior | PCL | 0.010 (0.002) | <0.001 | No path |  | 0.010 (0.002) | <0.001 |  |
|  | History of child abuse | 0.073 (0.034) | 0.031 | 0.021 (0.008) | 0.007 | 0.094 (0.034) | 0.006 |  |
| Adjusted (History of child abuse) | | | | | | | | |
| PCL-R | History of child abuse | 1.711 (0.631) | 0.007 | No path |  | 1.711 (0.631) | 0.007 | 0.021 |
| Lifetime self-harming behavior | PCL | 0.010 (0.002) | <0.001 | No path |  | 0.010 (0.002) | <0.001 |  |
|  | History of child abuse | 0.020 (0.034) | 0.557 | 0.018 (0.008) | 0.021 | 0.038 (0.035) | 0.277 |  |
